# Heat Shock Response and Heat Shock Proteins: Current Understanding and Future Opportunities in Human Diseases

**DOI:** 10.3390/ijms25084209

**Published:** 2024-04-10

**Authors:** Manish Kumar Singh, Yoonhwa Shin, Songhyun Ju, Sunhee Han, Wonchae Choe, Kyung-Sik Yoon, Sung Soo Kim, Insug Kang

**Affiliations:** 1 Department of Biochemistry and Molecular Biology, School of Medicine, Kyung Hee University, Seoul 02447, Republic of Korea; manishbiochem@gmail.com (M.K.S.); jac03032@khu.ac.kr (Y.S.); thdgus8543@khu.ac.kr (S.J.); sunheehan@khu.ac.kr (S.H.); wchoe@khu.ac.kr (W.C.); sky9999@khu.ac.kr (K.-S.Y.); 2 Biomedical Science Institute, Kyung Hee University, Seoul 02447, Republic of Korea; 3 Department of Biomedical Science, Graduate School, Kyung Hee University, Seoul 02447, Republic of Korea

**Keywords:** stress response, heat shock proteins, heat shock factors, thermotolerance, apoptosis, human disease

## Abstract

The heat shock response is an evolutionarily conserved mechanism that protects cells or organisms from the harmful effects of various stressors such as heat, chemicals toxins, UV radiation, and oxidizing agents. The heat shock response triggers the expression of a specific set of genes and proteins known as heat shock genes/proteins or molecular chaperones, including HSP100, HSP90, HSP70, HSP60, and small HSPs. Heat shock proteins (HSPs) play a crucial role in thermotolerance and aiding in protecting cells from harmful insults of stressors. HSPs are involved in essential cellular functions such as protein folding, eliminating misfolded proteins, apoptosis, and modulating cell signaling. The stress response to various environmental insults has been extensively studied in organisms from prokaryotes to higher organisms. The responses of organisms to various environmental stressors rely on the intensity and threshold of the stress stimuli, which vary among organisms and cellular contexts. Studies on heat shock proteins have primarily focused on HSP70, HSP90, HSP60, small HSPs, and ubiquitin, along with their applications in human biology. The current review highlighted a comprehensive mechanism of heat shock response and explores the function of heat shock proteins in stress management, as well as their potential as therapeutic agents and diagnostic markers for various diseases.

## 1. Introduction

In biological systems, cells and tissues are often exposed to several extreme environmental conditions or stressors, which cause acute to chronic stress, leading to physiological perturbations, cellular damage, and cell death. In response to stress stimuli, the cellular systems evoke adaptive mechanisms that help to defend against and to recover from the stress-induced insults. In fact, survival of a cell critically depends on its ability to cope with environmental as well as intracellular stress stimuli, and eventually depends on its association with the stress response. In response to a variety of stress stimuli, an adaptive and protective response is triggered, which depends on the duration and exposure of the stress stimulus delivered to the cellular system. The acquired adaptive capacity of a cell to stress ultimately determines the cell’s fate. Depending on the stress induction mechanism, two different pathways are involved; namely, the stress response and the high-temperature response [1]. The high-temperature response is an evolutionarily highly conserved process, exhibited by both prokaryotes and eukaryotes. It plays important role in a variety of normal cellular activities [2]. In 1962, for the first time the effect of temperature was observed by F. Ritossa in *Drosophila*. He observed induction of a specific set of puffs in the larval salivary gland polytene chromosomes of *Drosophila melanogaster* exposed to elevated temperature, termed as heat shock puffs. Further, Tissieres et al. demonstrated the mechanism of synthesizing a set of heat-inducible proteins in mammalian cells exposed to induced temperature [3]. McKenzie and Meselson characterized corresponding mRNAs transcribed at these heat shock puffs [4]. Later, Lindquist demonstrated the disappearance of pre-existing polysomes and the appearance of newly synthesized polysomes, encoded by an induced amount of heat shock proteins (HSPs), confirmed by the purified fraction of heat shock mRNA in situ hybridized to specific heat shock puffs [5]. The large number of studies focused on the molecular functions of heat shock proteins (HSPs) has revealed their crucial role in protein folding and maintaining proteo-homeostasis under normal and stress conditions, hence referred to as molecular chaperones [6]. This review highlights the significant features and functions of various HSPs in cellular homeostasis and human diseases, with a primary focus on the structure of major heat shock proteins, their co-chaperones, subcellular localization, and diverse roles in biological processes including cancer, aging, infections, immunity, and their implications for molecular diagnostics and therapeutics.

## 2. Stress Response and Heat Shock Factors (HSFs)

### 2.1. Heat Shock Response and Regulation

The heat shock response is triggered by different types of stressors, both biotic, and abiotic, such as from chemicals (like heavy metals, chemical toxicants, and oxidants), physical elements (such as heat, cold, and UV radiation), as well as environmental and biological agents (like fever, viral, and bacterial infections). These stressors cause various types of damage, both reversible and irreversible, at cellular and molecular levels. Such damage can alter the protein structure, leading to damage or degradation that affect cell functions [2]. When the heat shock response is initiated, protein transcription and translation are halted, possibly to reduce the burden of protein damage. This process is regulated by a subset of transcription factors known as heat shock factors (HSFs), which bind to the promoter region of the heat shock gene and induce its expression until the cells are normalized. In vertebrates, HSF1 mainly responds to heat shock or elevated temperature, while HSF2 and HSF4 are essential for development and differentiation processes (Table 1). HSF3 is an avian-specific transcriptional factor, co-expressed and co-activated with HSF1 by chemical and physiological stress [7,8]. HSF2 can modulate HSF1-mediated expression of heat-responsive genes under certain circumstances, suggesting that HSF2 can also participate in transcriptional regulation of the heat shock response [9]. HSF3 also interacts with other transcriptional factors, such as the MYB oncogene, directly and without stress [10]. Thus, HSFs play an essential role in regulation of the induced expression of heat shock proteins. At the translational level, these chaperones bind transiently and non-covalently to nascent polypeptides, and can unfold or unassembled proteins, aiding in protein biogenesis in two general ways: either by blocking non-productive protein–protein interactions or maintaining protein folding in their native state by sequestering folding intermediates, allowing coordinated folding by domains and assembly of oligomers [11]. These chaperones work in concert with co-chaperones and regulate the local protein folding and signaling network of the cell [12]. Overall, HSPs can be activated or induced by several stressors and protect the cell by influencing various cellular processes that determine cellular fate. Therefore, any external environmental agent or stimulus that triggers a transient cellular reaction with a consequent induction of transcription of a specific set of genes that are translated into proteins, the heat shock proteins, is collectively referred to as a heat shock response.

### 2.2. Heat Shock Transcription Factors and the Regulation of Heat Shock Response

**Figure 1:**
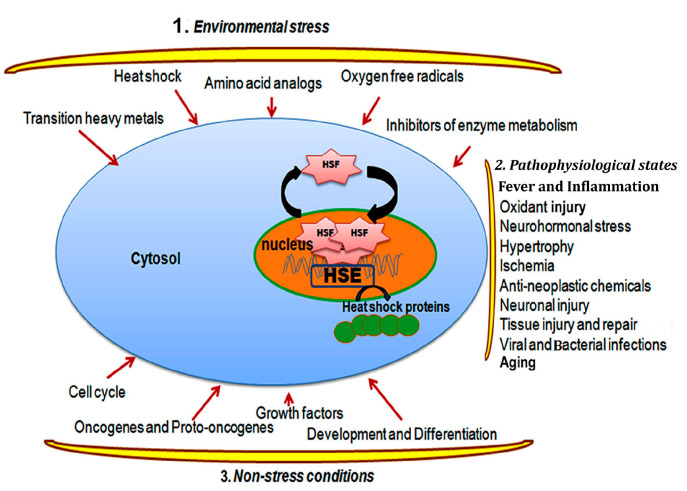
The diagram illustrates various stressors, including environmental stress, physiological stress, and physiological conditions, which trigger the activation and trimerization of HSFs. HSFs then translocate into the nucleus and activate the overexpression of stress genes such as heat shock proteins (HSPs).

The heat shock response is regulated both transcriptionally as well as post-transcriptionally. The transcriptional response to heat and other proteotoxic stresses is mediated by regulatory proteins, called heat shock transcription factors. These factors bind to the promoter region of the heat shock genes, known as heat shock transcription elements (HSEs) [2]. In vertebrates and plants, several different but related HSFs are known [8]. These factors are expressed ubiquitously and conserved from bacteria to humans. Yeast and *Drosophila* each have a single gene encoding HSF (yHSF and dHSF), which, when activated, causes transcription of heat shock genes [13,14]. Multiple heat shock factors are found in vertebrates, such as two in mice (mHSF1 and mHSF2) and three in chickens (cHSF1, cHSF2, and cHSF3) and humans (hHSF1, hHSF2, and hHSF4) [10,15,16,17,18,19] (Table 1). Among them, only HSF1 and HSF3 are involved in regulating the transcription of HSP genes in response to thermal stress. HSF2 and HSF4 are involved in unstressed conditions, and their levels are regulated by a wide variety of biological processes, such as immune activation and cellular differentiation [20]. High temperature activates HSF1 oligomerization and nuclear translocation, followed by enhanced DNA binding on HSP gene promoters. Among different vertebrate HSFs, HSF1 is mostly required for regulation of induced expression of HSPs [21,22]. A few reports provide evidence that mammalian HSF2 may also enhance heat-induced HSF1 activity. Avian cells lacking avian-specific HSF3 activity are defective in stress-induced HSP expression [23]. However, both HSF1 and HSF3 contribute to stress tolerance in birds [24]. In plants such as Arabidopsis, inactivation of both HSF1 and HSF3 genes results in significant impairment of the heat shock response [25]. HSF activation leads to its homo-trimerization, accompanied by acquisition of HSE DNA binding activity (Figure 1).

Several mutagenesis studies have shown a causal relationship between HSF1 oligomerization and HSE DNA binding activity. In yeast, HSF1 is constitutively present in a trimeric form with a high basal level DNA binding activity, which can be induced 10–15-fold by heat shock [26]. In most of the studies, the stressors and cell conditions that induced HSP expression have the potential to cause denaturation of cellular protein or synthesis of non-native proteins in case of amino acid analogs [27,28]. Most inducers, such as heat, can lead to the oxidation of both non-protein and protein thiols. This oxidation process typically results in the formation of glutathione-adduct or cross-links with proteins [29,30,31,32,33]. Therefore, accumulation of non-native proteins in the cell appears to be the likely initiating event that leads to induced HSP synthesis, mediated mainly via HSF1. Synthesis of HSPs are indeed involved in feedback regulation, which includes the synthesis of newly synthesized HSPs and disposal of stress-induced unfolded proteins. This stress response involved either refolding of the unfolded proteins or proteolytic degradation of unfolded/misfolded proteins. Further, re-association of HSPs and co-chaperones with their original cellular targets depends on HSFs regulation.

**Table 1:** Different types of heat shock transcription factors (HSFs), their localization and functions.

HSFs	Organism, and Homology	Oligomeric State, and Localization	Activators	Characterization	References
HSF1	Humans, mice, and chickens, 92% homology	Monomer (70 kDa), trimer (178 kDa), cyto-nuclear	Heat, metals, amino acid analogs	Constitutive and inducible, phosphorylation and developmental	[2,34]
HSF2	Humans, mice, and chickens, 92% homology	Dimer (127 kDa), trimer (202 kDa), cyto-nuclear	Hemin, embryogenesis	Activated during early blastocyst stage, limb buds, neuronal cells, and spermatogenesis	[2,20,35]
HSF3	Chickens and birds	Dimer, cytosol and nuclear	Heat, metals	Interact with cMyb and G1/S transition in cell cycle	[2,10,36]
HSF4 (a/b)	Humans	Trimer, constitutive, nuclear	Development	Active during lens development	[2,37,38]

Recent studies have indicated that HSF1, a transcription factor, is negatively regulated by the chaperone proteins HSP70 and HSP90. This suggests a negative-feedback loop for the regulation of HSP70 and HSP90 genes following heat shock response [2,20,39]. When cells are exposed to stress, this triggers the binding of HSF1 along with HSP90 and FKBP52 containing the chaperone complex. The ratio of multi-chaperone complex-bound trimeric HSF1 is influenced by a co-activator, DAXX [40]. DAXX interacts with several protein kinases and functions as an adaptor of apoptotic signal–regulating kinase1 that phosphorylates JNK [41,42,43]. Hence, there is precedent for the possibility that DAXX may direct a protein kinase to HSF1 that could phosphorylate the transcription factor or an associated protein. The transactivation of HSF1 requires its phosphorylation and SUMOylation, whereas deacetylation by NAD+-dependent Sirtuin (SIRT1) results in the attenuation of transcription of HSPs [20,36]. The kinases responsible for phosphorylation of HSF1 at several serine sites include glycogen synthase kinase 3*β* (GSK*β*) and c-jun N-terminal kinase (JNK) [32,44]. The cytokine interleukin 6 (IL-6) has been shown to further repress HSF1 by reducing the activity of GSK*β* [45]. However, a positive role of HSF1 phosphorylation in the stress-induced activation of HSP gene expression is also known. The exact mechanism of this effect has not been fully elucidated, although the protein kinase CK2 seems to be involved in enhancing transcriptional activity and the DNA binding of HSF1 by phosphorylating the threonine 142 residue [46]. Further, experiments have shown that HSF1-deficient cells have defects in HSP induction upon heat shock, which are more susceptible to apoptotic cell death due to exposure to heat shock [47]. In an experiment, mice lacking HSF1 showed elevated levels of tumor necrosis factor *α* (TNF-*α*), which resulted in increased mortality after endotoxin and inflammatory challenges [47]. Apart from these stress-regulating functions, HSF1 also modulates other genes, such as interleukin-1*β* and c-Fos [48,49], suggesting a role for HSF1 in regulating stress-responsive genes, other than those encoding HSPs. Recently, it was reported that HSF1 also functions in the circadian clock, as a circadian transcription factor. Through the use of a technique called differential display of DNA binding proteins (DDDPs), HSF1 has been shown to exhibit highly rhythmic transcriptional activity. Moreover, HSF1 enhances the expression of HSPs at the onset of the dark phase, coinciding with the period when animals become behaviorally active. Moreover, HSF1-deficient mice have a longer free-running period and thus display increased activity compared to their wild-type littermates. This suggests a dual role for HSF1 in both mammalian timekeeping and cellular protection systems [50].

Like HSF1, HSF2 is also involved in the regulation of heat shock protein gene transcription under non-stressful conditions. HSF2 exists in two isoforms, HSF2α and HSF2β, generated due to alternative splicing. While the HSF2α isoform is predominantly expressed in adult tissues, the HSF2β isoform is expressed in embryonic tissues. HSF2 DNA binding activity is high during early embryogenesis in tissues such as the heart, central nervous tissues, and testes [35]. The importance of HSF2 has been studied in the development of HSF2 null mice, which display a defect in gametogenesis and brain abnormalities with enlarged ventricles [51]. HSF2 has also been shown to bind to HSE on promoters of other heat shock genes, including HSP90 and HSP27, as well as the proto-oncogene c-Fos [52]. It is also known that HSF2 can form a heterotrimer with HSF1 in certain stress conditions [20]. Therefore, HSF2 is suggested to be important for constitutive as well as stress-inducible expression of HSE-containing genes.

Perhaps HSF1 and HSF2 have important roles in the heat-induced cellular response, but recent studies accentuate the role of other HSFs in the stress response. For example, avian HSF3 is described to be involved in cell cycle-dependent expression of HSPs. Studies have shown that HSF3/cMyb binding is involved in cell proliferation and G1/S transition of the cell cycle, along with the expression of HSP70, whereas binding of p53 tumor suppressor transcription factor to HSF3/cMyb dissociates HSF3 from cMyb, resulting in inhibition of HSP70 expression [10,36]. In contrast to other HSFs, the level of HSF4 expression is very low in many mammalian tissues, except lung and brain. Two isoforms of HSF4, HSF4a and HSF4b, are reported, but their differential effects are not yet characterized [53]. Recent studies have shown that HSF4 has a role in regulating lens-specific gamma-crystalline genes during lens development [37,38].

### 2.3. Functional Significance of Heat Shock Response in Thermotolerance and Environmental Adaptation

**Figure 2:**
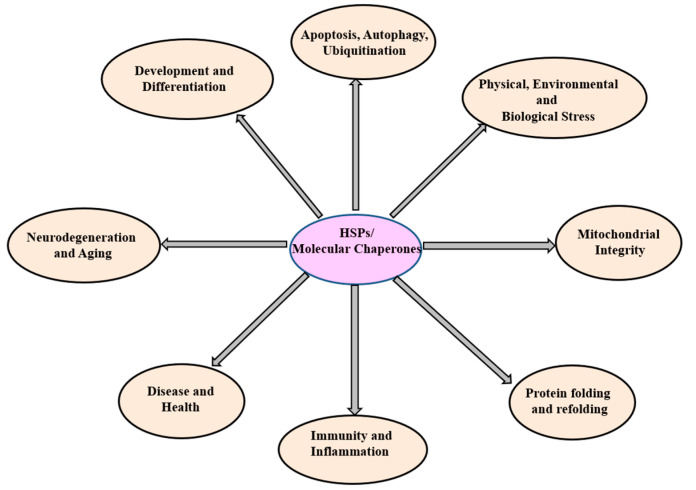
The diagram illustrates HSPs and their associated functions. HSPs (HSP100, HSP90, HSP70, HSP60, SHSPs, ubiquitin) oversee diverse cellular functions.

The phenomenon of heat shock response is highly conserved across all species from bacteria to higher eukaryotes. It relies on the degree of stress experienced by cells. When cells encounter different stressors, they promptly react by producing a set of proteins known as heat shock proteins. These proteins aid in maintaining protein homeostasis and confer stress response. However, prolonged exposure to these insults can lead to cellular damage and ultimately to cell death. The heat shock response protects the cells from various insults, including heat shock, harmful chemicals, toxic substrate, disease, and pathophysiological conditions such as ischemia, fever, inflammations, infection, and cancer [2,17,54,55,56]. HSPs are also involved in the cell cycle, cell proliferation, and cell differentiation. Based on the descriptions provided above, it appears that molecular chaperones have dual roles with interconnected pathways: either promoting cell survival or contributing to cell death (Figure 2). The equilibrium between these two opposing processes is contingent upon the cell’s ability to endure stress until it returns to its typical state.

The heat shock response protects cells from various deleterious effects induced by a broad spectrum of stressors, encompassing abiotic conditions such as high temperature, hypoxia, osmotic imbalance, heavy metals, UV radiations, as well as biotic stresses such as viruses, bacteria, parasites, fungi, harmful insects, and weeds [57,58,59,60,61,62,63,64,65,66,67]. HSPs have undergone extensive study, with their structure and function characterized in most insects and invertebrates. The majority of HSP genes are upregulated during various developmental stages, including embryonic, larval, pupal, and adult stages [68]. In *Sarchophaga crassipalpis*, the expression of HSP23 and HSP70 is upregulated during pupal diapause [69]. Conversely, HSP90 was found to be downregulated two days after pupation, while HSP23 and HSP70 transcripts were unregulated shortly after onset of diapause, specifically five days after pupariation [70]. This suggests distinct regulation of HSPs in response to thermal injury experienced by diapausing pupae [69]. The level of HSP60 mRNA is differentially regulated in development of insects such as *Drosophila*, with the HSP60 mRNA being overexpressed in the early stage of the embryogenesis as compared to all subsequent stages, following which the mRNA level decreases [71].

Increased expression of small heat shock proteins (sHSPs) has been observed to extend the lifespan of *Drosophila* and increases thermotolerance [72]. High thermal resistance has also been found in-vitro and in-vivo experiments on the silkworm, *Bombax mori*, with induced expression of HSPs reported after a high-temperature stress. In-vivo induced expression of HSPs has also been observed in different insect cell types [73]. Mutational studies on *Drosophila* (Mnn1) have shown it is hypersensitive to several stressors and exhibits increased genomic instability when subjected to high-temperature stress [74]. However, *Drosophila* cell cultures have shown increased expression of sHSP transcripts in northern hybridization, induced by high temperature or by exposure to physiological doses of insect molting hormone, ecdysterone [75]. Thus, sHSP regulates the developmental changes in the transcription and chromatin structure in *Drosophila* at high temperatures, required for acclimatization to extreme temperatures and other stressors [75]. 

Chaperonin (*cpn60*) is an important heat shock protein, present in all forms of life. It assists in the folding, assembly, and transport of various cellular proteins and is known to be present in both the cytoplasm and mitochondria [76]. HSP60 plays a role in diverse cellular functions, particularly in transporting proteins from the cytoplasm to the mitochondria. In apoptosis-mediated cell death, HSP60 performs a dual role. While residing in the mitochondria, it acts as an anti-apoptotic factor; however, in cytoplasm, it regulates the pro-apoptotic process. Hence, it is linked to several human pathologies [77,78,79]. As a chaperonin, it also resides in subcellular organelles such as chloroplast and endosymbiotic origin cells; however, its functions in these organelles remain unexplored.

HSP60 serves many functions, and may be present intracellularly, extracellularly, or even on the cell surface. Its presence on the cell surface has been reported in normal, stressed, and tumor cells, and is thought to be associated with membrane transport and signaling [80]. An increase in surface HSP60 levels has been reported to lead to the activation and maturation of dendritic cells, resulting in an anti-tumor T cell response [81,82]. Thus, HSP60 surface expression indicates a danger signal in response to host innate immunity. Mouse and human macrophages and endothelial and smooth muscle cells were found to elicit a pro-inflammatory response when incubated with recombinant human HSP60. Interestingly, microbial HSP60/65 also induced a pro-inflammatory response in innate immune cells. This suggests that damaged autologous cells and microbial pathogens may alert innate immunity via the same recognition system [83,84,85].

## 3. The Heat Shock Proteins (Molecular Chaperones)

The cell responds to stress by producing a unique set of proteins known as heat shock proteins (HSPs). HSPs are present in all cells and are even expressed in normal conditions, acting as molecular chaperones [86]. These stress proteins are characterized based on their molecular weight into several groups, such as high molecular weight heat shock proteins (such as HSP100, HSP90, HSP70, and HSP60), sHSPs (12–40 kDa), and ubiquitin (8–12 kDa) Table 2. Researchers follow the guidelines described by Kampinga et al. (2009) in naming HSPs. HSPs help maintain cellular protein homeostasis and assist in the folding, assembly, and disassembly of protein complexes [87]. They also inhibit improper protein aggregation resulting from thermal stress, degradation, and repair, synthesis of naïve polypeptide folding [88], assembly and trafficking, and activation of the immunological system in response to viral and bacterial infections [89,90,91] (Table 3).

Molecular chaperones play important roles in cells, including proper folding of native proteins, protection of protein from improper folding, and maintenance of their functions [92]. HSPs respond rapidly to stressors and their levels normalize once cell conditions are restored. The induction of HSPs relies on ATP binding and hydrolysis in affected cells, facilitating their chaperone functions. HSP60 functions are activated by ATP-binding and hydrolysis in the mitochondria. Other HSPs are localized in specific organelles, such as GRP78 in endoplasmic reticulum (ER), which regulates protein assembly and trafficking to their respective cells, and responds to ER stress, and UPR to suppress protein processing when ER stress is induced [93]. In this review, we explored the functions of HSP60, HSP70, HSP90, HSP100, one representative of the sHSP (HSP27), and ubiquitin–protein in various conditions, including thermal-stress, protein folding, and in different human diseases.

### 3.1. HSP100 (HSPH; Clp Family) and Functions

**Figure 3:**
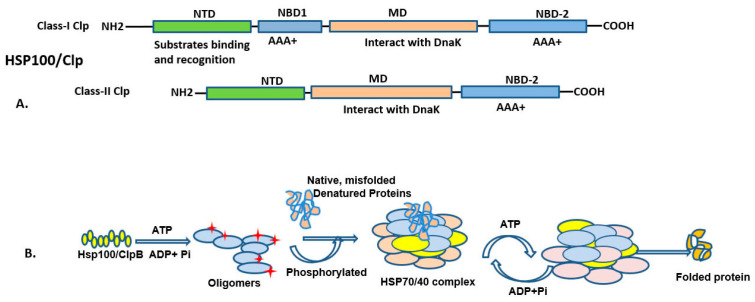
The diagram illustrates the HSP100/Clp protein domain arrangement (**above**) and HSP100 assisting protein folding (**below**). (**A**) HSP100/ClpB comprises three main domains, the substrate binding domain, middle domain, and C-terminal domain, as illustrated in Class-I and Class-II. (**B**) It facilitates the folding of nascent polypeptides and misfolded proteins in an ATP-dependent manner. ATP is required for activating protein folding and binding of HSP70/40. Upon proper folding of protein, ATP is hydrolyzed, leading to dissociation of the HSP70/40 complex, and the folded protein is released into the cytoplasm.

HSP100 chaperones, also known as Caseinolytic protease (Clp) proteins, are members of the AAA+ superfamily, ATPase associated with various cellular activities. They comprises nucleotide-binding domains (NBDs), as well as a number of functional motifs referred as Walker A and Walker B motifs respectively [94,95]. HSP100 consists of three main domains: the N-terminal domain, regulatory M-domain, and ATP-binding domain (Figure 3). Two of these classes depend on ATP-binding sites; class I consists of ClpA, ClpB, ClpC, ClpD, ClpE, and ClpL, and class II consists of ClpX and ClpY [96]. In *E. coli*, Clp peptidase consists of two identical heptameric rings, which enclose a large central cavity containing protease active sites. The ClpP peptidase consists of two heptameric rings, which enclose a large central chamber with two small axial pores. In the inner surface of the Clp hexamers, 14 active serine sites are sequestered [97]. Clp forms a complex with ClpX and ClpA to generate ClpAX or ClpXA proteases, and degrades the protein substrate. In these complexes, ATP is required for multimerization of the ATPase for unfolding and chaperone activity, and degrades the protein substrate [98,99,100]. In *E. coli*, ClpA, the tripeptide sequence (617–619 aacs) is IGL and is in a 28-residue region that substitutes for the HslU loop. Further, studies have revealed that HsIU (ClpY) does not interact with ClpP ATPase [101]. Recent studies have shown that HslU does not interact with Clp, but interacts with other peptidases, called HslV (ClpQ) [102]. ClpB has been extensively studied in bacteria such as E.coli, and B. subtilis, and the yeast cytosolic and mitochondrial homologs HSP104 and HSP78 respectively, along with plant homologue HSP101 are a well-characterized chaperones [103]. ClpC (mecB) protein showed heat-inducible activity and help in survival of bacteria subjected to stressors such as ethanol and high salt [104]. HSP100/Clp chaperones are most extensively expressed and studied in bacteria and yeast, and thus could be promising targets for developing novel antibacterial strategies, which aid development of drugs against antibiotic resistance of pathogenic bacteria.

The HSP104/ClpB M-domain binds to the cytoplasmic HSP70 chaperone and HSP40 for protein disaggregation [105,106,107], while the toxicity of HSP104 variants is regulated by its interaction with HSP70 [107]. Due to their protein disaggregation and degradation properties, ClpB and its yeast ortholog HSP104 are being developed for novel therapies, targeting protein aggregation diseases in humans, such as Alzheimer’s disease, Parkinson’s disease, and Huntington’s disease (Table 4) [108]. Since many Clp proteins are not reported in humans, the functions of HSP100 in survival and virulence in pathogenic bacteria are being investigated for their role in regulating virulence. Consequently, inhibitors of HSP100/Clp could potentially serve as targets for addressing many pathogenic bacterial infections.

### 3.2. HSP90 (HSPC) and Functions

**Figure 4:**
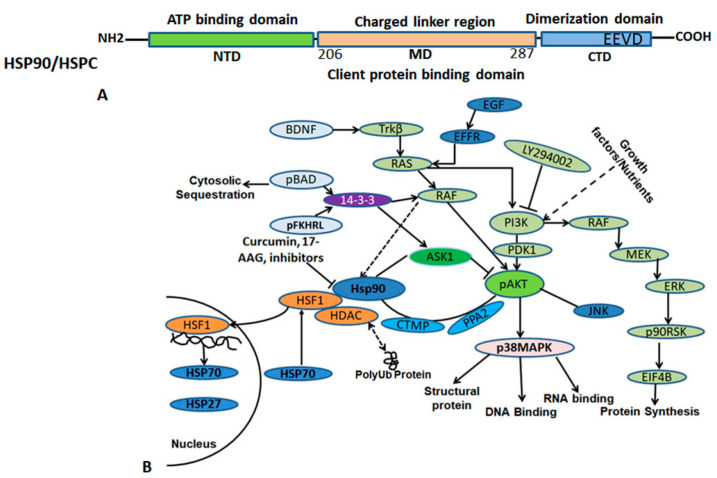
The diagram illustrates the HSP90/HSPC protein domain arrangement (**above**) and HSP90 interacting client proteins (**below**). (**A**) HSP90/HSPC consists of three main domains, the ATP binding domain, middle domain, and C-terminal domain. The C-terminal domain contains highly conserved sequences that help in dimerization. (**B**) HSP90 has many interactors that bind to the middle domain (206–287 amino acids) region and regulate various functions in the cells. Solid ‘→’ shows direct interaction, doted ‘→’ intermittent function.

HSP90 is a highly abundant molecular chaperone, expressed ubiquitously in eukaryotes, essential for regulating the activity of numerous client proteins. In normal conditions, it makes up 1–2% of cellular protein. HSP90 plays a crucial role in various cellular processes, including signal transduction, protein folding, protein degradation, cell proliferation, differentiation, and apoptosis [109,110,111,112]. HSP90 aids in signal transduction by ensuring the proper folding of polypeptides and maintaining active states of substrates, such as transcription factors and protein kinases [113]. There are two known isoforms of HSP90, the inducible and non-inducible forms. Additionally, there is another isoform of HSP90, called HSP90N, which is associated with cellular transformation [114]. Grp94 (94 kDa glucose-related protein) is present in endoplasmic reticulum in all eukaryotes except fungi. The mitochondrial homolog TRAP (TRAP1/2, tumor necrosis factor receptor-associated protein) is present in the mitochondrial matrix and closely related to the eubacterial homolog of HtpG [115]. The HSP90 polypeptide comprises more than 700 amino acid residues and is divided into three functional and structural domains [113,116]. It has an amino-terminal (N) domain that contributes to ATP binding [117], a charged linker region, a middle (M) domain, and a C-terminal that is involved in dimerization. The tertiary structure of HSP90 has been determined by X-ray crystallography [118,119]. This domain has a chaperone activity, which aids unfolded peptides in an ATP-dependent manner [119,120]. The intermediate domain or middle domain (M) comprises a highly charged hinge region (amino acids 206–287), and is associated with the binding of client proteins (Figure 4). This highly charged region increases substrate affinity and is required for chaperone activity [111,121]. Moreover, it contributes to co-chaperone interaction and client protein binding, making it crucial for HSP90′s full functional activity. Interestingly, this region is absent in HtpG, an HSP90 homolog of *E. coli*; however, its role in divergence of HSP90 evolution remains unexplored. It can be deleted without affecting the growth of yeast [110,122]. Recently, the middle domain has been shown to contribute to co-chaperone interaction and binding of client proteins [121]. The C-terminal domain contributes to dimerization of HSP90 [123]. The C-terminal domain exhibits different substrate specificity compared to the N-terminal domains, making it an important domain to consider [124]. While both N- and the C-terminal domains independently suppress aggregation of fully denatured polypeptides, the full length is required to refold partially denatured substrates [125]. HSP90 is a protein, which works with other co-chaperones to perform its full functional activity. Its main function is to help newly synthesized proteins fold correctly and also to stabilize and refold denatured proteins under stress. HSP90 binds to a large number of client proteins, with the co-chaperone requirement varying depending on the client. Most of these client proteins are involved in signal transduction [126].

HSP90 plays a crucial role in the development of cancer and drug resistance. It is found to be highly expressed in many cancers. Natural inhibitors, pharmacological inhibitors, and small molecule inhibitors, such as curcumin, geldanamycin and its derivatives 17-allylamino-17-demethoxygeldanamycin (17AAG) and 17-dimethylaminoethylamino-17-demethoxygeldanamycin (17DMAG), are effective in treating hematological cancer and solid tumors [127,128]. These inhibitors destabilize and degrade several client proteins of HSP90 that are mutated and activated in various cancers. Survivin, an oncogene, binds to HSP90 in tumor cells, stabilizes itself from proteasomal degradation and protects the cells from apoptosis [129]. In vivo studies have shown that targeting HSP90 co-chaperones such as Cdc37 to prevent kinase aggregation may also be relevant to neurodegenerative disease-related pathways. Celastrol and Withaferin A are more effective in disrupting the HSP90/Cdc37 complex in cells, along with targeting other pathways in the cells [130,131,132]. In neurodegeneration, several HSP90 co-chaperones, such as ATPase homolog 1 (AHA1), the peptidyl-prolyl cis-trans isomerase FKBP family, and HSP-organizing protein (HOP), are overexpressed with age, contributing to disease progression. AHA1 overexpression facilitates tau fibril formation, while FKBP52 interferes with the binding of AMPA receptors, which induces synaptic plasticity [133,134]. Moreover, HOP overexpression exacerbates α-synuclein toxicity in Parkinson’s disease (PD) [135] and amyloid toxicity in Alzheimer’s disease (Table 4) [136]. Inhibition of HSP90 results in induced expression of HSP70, which negatively regulates the activity of HSF-1s. Various HSP90 inhibitors such as SNX-0723 reduced the α-synuclein toxicity in PD via induction of the HSP70 level in a PD rat model, indicating it as a promising candidate to protect against neurodegeneration [137,138]. Other inhibitors, such as Platycodin D (PD), disrupt the interaction between HSP90 and Cdc37, leading to subsequent degradation of multiple HSP90 client proteins, without triggering the feedback increase of HSP70 [139]. Similarly, Kongensin A, has also been evaluated in vitro for inhibiting HSP90 and Cdc37 interaction, which prevents the RIP3-dependent necroptosis and activates apoptosis in various cancer cell lines. Therefore, it could potentially serve as candidate for anti-cancer therapy [140]. Many HSP90 inhibitors regulate the client chaperone interaction, although more investigation is required to explore their functions in other human diseases. HSP90 inhibitors have been tested for developing drugs for cancer and neurodegeneration. However, the off-target effect of HSP90 might be crucial to both the disease and normal cells. Therefore, further investigations are necessary to standardize the effective concentration of HSP90 inhibitors with minimal side effect on normal cells for therapeutics [141].

### 3.3. HSP70 (HSPA/B) and Functions

HSP70 is a ubiquitously expressed molecular chaperone highly conserved across all organisms. It is a 70 kDa protein involved in multiple processes. HSP70 is involved in protein folding of newly peptides, refolding of misfolded and aggregated proteins, protein transportation, assembly and disassembly of protein complexes, protein transportation, and degradation of non-functional proteins [142,143]. The HSP70 family of proteins consists of two forms; a constitutive 73 kDa protein (HSP73), and a stress-inducible 72 kDa protein (HSP72). HSP72 is induced in most cells after exposure to different types of biological and environmental stressors. The nuclear form of HSP72 is involved in protection against different types of environmental stresses, whereas the extracellular HSP72 functions as a signaling molecule in the immune system. In eukaryotes, HSP70 is present in cytoplasm, and the nucleus. In other organelles such as the endoplasmic reticulum and mitochondria, HSP70 members are also present [143].

**Figure 5:**
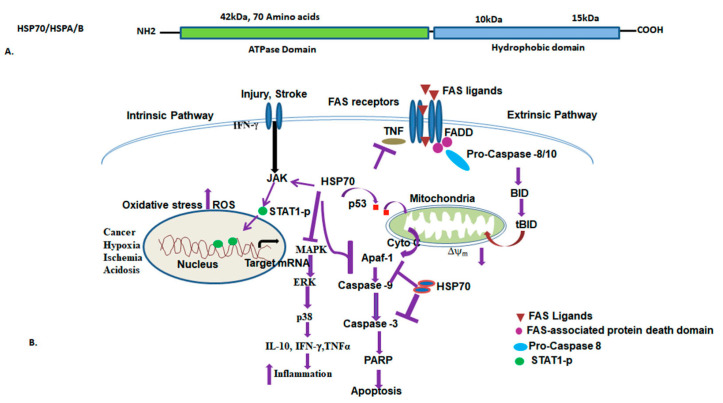
The diagram depicts the domain arrangement of the HSP70/HSPA/B protein (**above**) and its associated functions (**below**). (**A**) HSP70 comprises two main domains: the ATPase domain and the C-terminal domain. ATP is necessary for activating protein folding by binding to the ATPase domain. (**B**) HSP70 is implicated in various biological functions. The diagram illustrates its involvement in cellular pathways such as mitochondrial integrity, apoptosis, protein folding, inflammatory responses, and disease regulation. It also represents HSP70’s interactions with other cellular proteins such as p53, TNFα, MAPK, and Apaf-1, which regulate cancer, hypoxia, inflammation, and apoptosis. Loss of the mitochondrial membrane potential leads to apoptotic cell death. Green circle: phosphorylated-STAT1; Marron triangle: FAS-ligands, Violet circle: Fas-associated Protein death domain; Sky-blue oval: Pro-caspase-8; Red box: p53.

The HSP70 protein is made up of two different domains: a 40 kDa N-terminal domain, and a 25 kDa C-terminal domain. The N-terminal domain binds and hydrolyzes ATP, while the C-terminal domain consists of a hydrophobic domain that binds to five amino acid segments of the unfolded target proteins or client proteins [144]. These domains are joined by a flexible linker. To function efficiently, HSP70 requires a co-chaperone consisting of a J-protein and nuclear exchange factor. The J-protein, also known as *E. coli DnaJ*, regulates the binding of HSP70 to its client protein. The binding of HSP70 to the client protein depends on the ATP hydrolysis and nuclear exchange factors [87]. The on and off rates of binding of client proteins depend on the ATPase activity of the N-terminal domain [145]. The ATPase activity is stimulated by J protein, which facilitates the binding of the client protein (Figure 5). Conversely, NEF binding stimulates dissociation of ADP, results in dissociation of client protein and consequently, recycling of the HSP70 molecule [146]. The J family is known as HSP40, with a highly conserved ~70 amino acid signature region. The J domain consists of conserved His, Pro, and Asp tripeptides (HPDs) that stimulate the ATPase activity of HSP70 [147,148]. The conformation and function of HSP70 are tightly regulated to allosteric mechanisms and binding of the nucleotide and substrate [149]. HSP70 is expressed constitutively in a variety of cells and is induced by a variety of stressors, including hypoxia, ischemia, acidosis, energy depletion, cytokines such as tumor necrosis factors α (TNF-α), and UV radiation.

Among the HSP70 family proteins HSP72 (HSP70), HSP73 (HSC70), HSP75 (mHSP70), and HSP78 (Grp78), HSP72 is the most heat-inducible and it is synthesized in response to multiple stressors. Research has shown that HSP70 is involved in a variety of human diseases, including cancer, neurodegeneration, aging, and infections. In cancer, an isoform of HSP70 called HSPA9 binds directly to p53, inactivates it, and degrades it through proteasomal degradation pathways. Another isoform, HSPA8, interacts with p53 and stabilizes the inactive p53 mutant, promoting cell survival [150]. Studies have also shown that whole-body hyperthermia resulting from dysfunction of some critical target tissues can lead to morbidity and mortality [151,152,153]. Although HSP70 is involved in several intracellular functions, the extracellular localization has been found to have an immunological role in tumors by stimulation of effector cells of the innate immune system [154,155]. HSP70 also plays an anti-apoptotic role by prevention of JNK-induced phosphorylation and inhibition of Bcl-2 and Bcl-xL anti-apoptotic proteins, apoptosis protease activating factor 1(APAF-1), and apoptosis-inducing factor (AIF), thereby promoting cell survival through the maintenance of mitochondrial stability [156]. Overexpression of HSP70 can remove these proteins from their signaling pathways, and promote cell survival. Additionally, HSPA8 degrades tumor necrosis factor alpha (TNF-α) in a CHIP-dependent mechanism, inhibiting apoptosis [157]. In viral infections, the HSP70 isoform HSPA8 helps in replication of viral genes. Inhibition of HSP70 can result in the degradation of the capsid protein in the ZIKV virus. Further, investigation is required to determine how HSP70 inhibition could be implicated in other viral infections, such as hepatitis B, rotavirus, Epstein-Barr, HIV, and respiratory syncytial viruses [158,159,160,161]. Furthermore, HSP70 functions are also studied in aging and neurodegeneration. A decreased level of HSP70 in the human brain cortex and hippocampus facilitate accumulation of damage and unfolded proteins which lead to proteotoxicity [162,163,164]. Additionally, reduced levels of HSP70 have been reported in various other human diseases such as pulmonary fibrosis, various types of myopathies, cholesterol sphingolipidosis, diabetes, and obesity [165,166,167,168]. HSP70 expression is reduced in obesity, diabetes, and insulin resistance. HSP72 inhibits the activation of stress kinases such as c-jun-NH2-terminal kinase (JNK), which promotes insulin resistance by inhibiting the phosphorylation of insulin receptor substrate 1 (IRS-1), thereby regulating T2DM [169]. Moreover, HSP72 exhibits anti-inflammatory effects in cells, which are elicited by suppression of TNF-α, IL-1β, and IL6. Furthermore, HSP70 is involved in regulating oxidative stress and inflammation in the blood vessels. Therefore, the level of HSP70 may serve as a useful indicator in controlling atherosclerosis and evaluating the preventive effect of atherosclerotic cardiovascular disease (Table 4) [170]. Additionally, the endogenous HSP70 could serve as a promising marker for early detection of various diseases, particularly in prostate disease and bronchopulmonary dysplasia (BPD) [165,171]. In vivo intranasal administration of recHS70 ameliorates the amyloid β level and amyloid plaques [172]. Similar results have also been corroborated in diabetic mouse models, indicating increased sensitivity to insulin [173]. Intracellular HSP70 levels are induced in various cancers and exhibit anti-apoptotic functions by preventing the apoptosis of cancer cells [174]. Consequently, HSP70 inhibitors are employed in various cancers, but their specificity with different isoforms poses a major challenge in selecting them for human diseases. Further investigations are required to select the specific HSP70 isoform that is differentially expressed in tumor or cancer types that need to be evaluated for therapeutics.

### 3.4. HSP60 (HSPD/E) GroEL/ES and Functions

**Figure 6:**
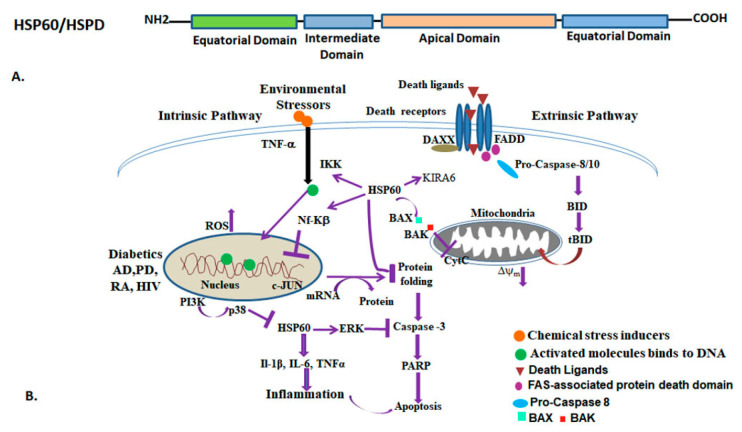
The diagram depicts the HSP60/HSPD protein domain arrangement (**above**), and its activation and functions (**below**). (**A**) HSP60/GroEL comprises three main domains, the equatorial domain, intermediate domain, and apical domain. (**B**) The diagram illustrates the involvement of HSP60 in various biological processes. It delineates different cellular pathways such as mitochondrial integrity, apoptosis, protein folding, inflammatory responses, and disease regulation associated with HSP60. HSP60 interacts with other cellular proteins such as BAX, NF-kβ, IKK, and p38 to regulate cancer, diabetes, neurodegeneration, inflammation, and apoptosis. Various stressors and chemical and physical activations of downstream signals regulate protein folding and cell death pathways. Mitochondrial dysfunction and loss of membrane integrity lead to activation of cell death via apoptosis. Abbreviations: Alzheimer’s disease (AD), Parkinson’s disease (PD), Human immunodeficiency virus disease (HIV), Rheumatoid arteritis (RA). Red circle: Chemical stress inducers; Green circle: Activated molecules bind to DNA, Marron triangle: Death ligands; Violet circle: Fas-associated Protein death domain; Sky blue oval: Pro-caspase-8; Green box: BAX; Red box: BAK.

HSP60 is a mitochondrial stress protein known as molecular chaperonin. It is a nuclear-encoded protein, synthesized in the cytoplasm and transported into the subcellular organelles such as mitochondria and endoplasmic reticulum. HSP60 is present in both the mitochondria and cytoplasm, with 80–85% being mitochondrial and the remaining 15–20% in the cytoplasm [175,176]. The HSP60 family includes the *E. coli* homolog GroEL/ES, Cpn60, human homolog CCT, TRiC (HSPD/E), and mitochondrial HSP60 (mtHSP60). HSP60 is divided into two groups, group I and group II chaperonins [144,177]. The absence of group I chaperonin in archaea is intriguing since the HSP70 (Dnak) chaperonin machine is present in a single organism [178]. The archaeal chaperonin system is cylindrical in structure, similar to bacterial GroEl/ES. Subsequently, in *E. coli,* the native polypeptide reaches the GroEL/ES system for final folding [120,179,180]. An archaeal chaperonin complex resembles the eukaryotic cytosolic protein, called TCP1 (tailless complex polypeptide-I), CCT (chaperonin containing TCP-1), or TRIC (TCP-1 ring complex) [181,182]. The chaperonins specific to organelles are believed to have diversified from the genome of the primitive bacteria after endosymbiotic events [183,184]. HSP60 forms a double heptameric rings of identical ~57 kDa subunits, assembling into a hollow-core structure, stacked back-to- back. Each subunit consists three domains; a equatorial domain housing the ATP binding sites, an intermediate domain, and an apical domain that binds to both substrate and GroES. Normally, the GroEL/ES complex in prokaryotes corrects the folding of post-translated folding/assembly of subunits of certain major oligomeric proteins that depend upon their association with GroES, a heptameric ring of identical 10 kDa subunits, which binds to the apical GroEL domains and forms an asymmetrical complex [185,186,187]. In prokaryotes, the GroEL/ES complex corrects the folding of post-translated folding/assembly of subunits of certain major oligomeric proteins that depend upon their association with the chaperonin, which are themselves not part of the assembled complex [6]. In *E. coli*, the GroEL monomer unit comprises three structural domains: A (apical), I (intermediate), and E (equatorial). The equatorial domain resides at the base of the ring, while the apical domain forms the barrel, and the intermediate domain connects the other two domains. Since the intermediate domain is smaller than the other two domains, it creates open spaces or windows that connect the outside surface to the inner cavity of the barrel. On the other hand, GroES is a single ring composed of 7-subunits and functions as a co-chaperone of GroEL. The eukaryotic cytosol chaperonin CCT belongs to type II system, a member of group II. Its structure resembles that of the GroEL/ES complex but is constructed with eight different subunits per ring, ranging from 50 kDa to 68 kDa (forming a heteropolymer ring) [120,188,189]. In addition to morphological differences, group I and group II chaperonins differ in functional aspects too. CCT can bind to and mediate ATP-dependent folding of actin and tubulin, among other substrates. In archaea, the chaperonin consists of two subunits similar to other eukaryotes. The archaeal chaperone subunits form both homo- and hetero-polymeric rings. Two rings stack end to end to form a barrel (cavity) that is assumed to be a peptide folding cage. An extensive study was carried out in *T. acidophilum* [190], an archaea comprising two domains with alternating subunits, namely, α and β, of ~58 kDa in size. Conformational changes take place in the native proteins enclosed inside the cage, transitioning from hydrophobic to hydrophilic states, initiated by ATP binding (Figure 6). The enclosed polypeptide is folded correctly and released as the GroES ring is displaced from the GroEL. Another example, *H. valcanii*, an extreme halophile, has homologous genes named cct1, cct2, and cct3, which are akin to the eukaryotic CCT gene, and non-heat inducible. In a cis-configuration, a lining of highly charged residues potentially plays a functional role in substrate interaction. However, in a trans-confirmation, conserved charged residues belonging to domain A and I, buried between monomers, are involved in hydrophobic contact, thereby facilitating the interaction of charged residues with substrates [191,192]. Multiple alignments of HSP60 protein from different species have revealed that glycine (G) is the most conserved residue and can be a “filler” or “hinge” for backbone conformations of GroEL/ES [192].

HSP60 is predominantly found in the mitochondria; however, some evidence has shown that it can also be transformed into the nucleus and on the cell surface [79,193,194]. However, the role of HSP60 in the nucleus remains unexplored and requires further investigation. The primary function of HSP60 is to assist in the folding of newly synthesized proteins and the refolding of denatured proteins. In addition, HSP60 is involved in various other functions such as regulating stress, maintaining mitochondrial integrity, immune response, and pro- and anti-apoptotic functions. Epitopes originating from mycobacterial HSP65, identical to human HSP60 peptides, elicit cytotoxic T cell responses in healthy individuals [195]. Studies have shown that T cells reactive to self-HSP60 are associated with spontaneous remission in juvenile idiopathic arthritis and confer resistance to experimental arthritis in Lewis rats [196,197]. T cells targeting HSP60 and secreting IL-10 may have a beneficial role in reducing inflammation during autoimmune diseases but could be detrimental in the context of infections [197,198,199]. Conversely, T cells responding to HSP60 by producing proinflammatory cytokines may contribute to tissue damage in autoimmune conditions but could aid in combating pathogens [200]. Additionally, antibodies targeting self-HSP60 are detected in several autoimmune diseases, including rheumatoid arthritis (RA), lupus, inflammatory bowel disease, and atherosclerosis. Cytosolic HSP60 is involved in cell signaling in different cell types, such as cardiac myocytes and hepatocytes [201]. It has been observed that cytosolic HSP60 interacts and regulates the activation and phosphorylation of NF-kB kinases (IKK) to protect the cells from mitochondrial-derived oxidative stress, mediated by NF-kB-targeted gene expression [202,203]. HSP60 family proteins have been implicated in human diseases such as type 2 diabetes, hepatitis B, cardiovascular disease, atherosclerosis, periodontitis, juvenile idiopathic arthritis, and various cancers (Table 4) [204,205,206,207]. In diabetic patients, HSP60 modulates the TLRs and IGF-I receptor level linked with reduced expression of HSP60 in T2DM [208]. HSP60 family proteins have also been implicated in cell signaling, endothelial stress activation, and cardiovascular diseases via TLRs. In most vascular diseases, TLRs and HSPs regulate cellular signaling. Among 10 human TLRs, TLR2 and TLR4 are reported in the pathogenesis of cardiovascular diseases [209,210]. HSP60 is involved in stimulating vascular smooth muscle cell migration, via the TLR4 and ERK/p38 MAPK pathways, which are key contributors to atherosclerosis. This study indicates that HSP60 expression serves as a potent danger signal to the immune system, triggering the generation of IL8, which aids in managing infections and diseases [211]. The HSP60 level is context-dependent and also depends on the types of cancer and tissues as compared to normal tissue, thus targeting HSP60 may be a potential candidate to reduce both the adverse effects and drug resistance in many tumor and cancer cells [212,213]. HSP60 also plays an important role in both innate and adaptive immunity. HSP60 plays a role in diabetes-induced neuroinflammation by activating microglia and astrocytes that stimulate production of pro-inflammatory cytokines IL-1β, IL-6, and TNF-α through different signaling pathways such ERK-1/2, JNK, and NF-κB [214]. Another study reported that HSP60 prevents inflammation-induced cell death in rheumatoid arthritis (RA) via secretion of anti-inflammatory cytokines IL-4 and IL-10 at the inflammation site in the bone [215,216]. Studies have shown that HSPs contribute to protein metabolism and the aggregation of both the Aβ and tau [217]. HSP60 also regulates Aβ accumulation and aggregate formation in the neurons [218]. It has been observed that human HSP60 (HSPD1) binds to Aβ oligomers and reduces neurotoxicity by inhibiting its interaction with the membrane [219,220]. In vitro, mitochondria HSP60 binds to Aβ oligomers, and reduces Aβ-mediated mitochondrial dysfunctions and neuronal death [221]. Thus, the above discussion indicates that HSP60 plays a crucial role in development and is also important in the regulation of many diseases such as cancer, atherosclerosis, and neurodegeneration. This may further aid the development of early markers for diagnosis and treatment in various diseases.

### 3.5. Small Heat Shock Proteins (HSPB)

**Figure 7:**
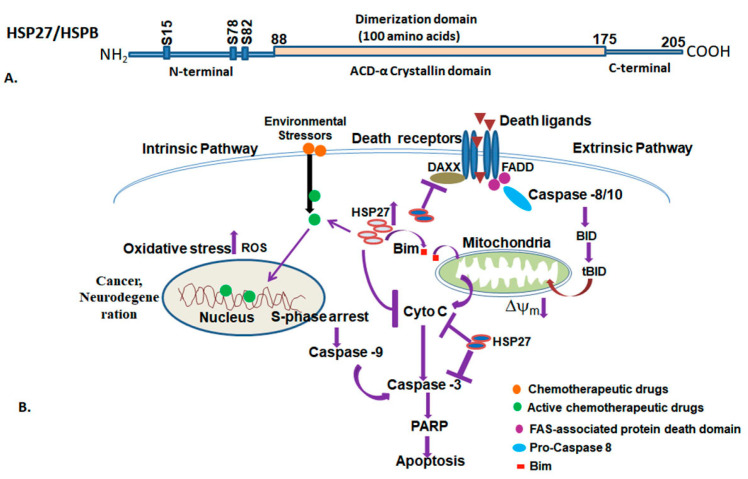
The diagram illustrates the domain arrangement of the HSP27/HSPB protein (**above**), and HSP27-associated functions (**below**). (**A**) HSP27, comprising the N-terminal domain, α-crystallin domain, and C-terminal domain. (**B**) The diagram depicts the HSP27 involved in various cellular pathways. The diagram illustrates HSP27 involved in various crucial processes including mitochondrial integrity, apoptosis, protein folding, and cell cycle regulation. HSP27 interacts with cellular proteins such as Bim, cytochrome C, and DAXX, thereby modulating processes regulated to cancer, neurodegeneration, and apoptosis. Various stressors and chemical and physical stimuli activate downstream signals that regulate protein folding and cell death pathways. Dysfunctional mitochondria and compromised membrane integrity trigger cell death through apoptosis. Red circle: Chemotherapeutic drug; Green circle: Active chemotherapeutic drugs; Marron triangle: Death ligands, Violet circle: Fas-associated Protein death domain; Sky-blue oval: Pro-caspase-8; Red box: Bim.

Small heat shock proteins (sHSPs) are expressed ubiquitously across all organisms, including plants. These proteins have low molecular weights, typically ranging from approximately 16 kDa to 42 kDa [120,222]. In contrast to other families of HSPs, the small HSPs family exhibits a lower level of sequence conservation at both nucleotide and amino acid levels. Small HSPs display significant diversity within species groups, with intra-specific variations being less pronounced compared to differences between species [222,223]. The family of small heat shock proteins currently consists of 11 members that are characterized by the conserved crystalline domain, flanked by variable N- and C-termini. Among them, HSP27 (HSPB1), αA crystalline (HSPB4), and αB-crystalline (HSPB5) are extensively studied. The α-crystalline domain is formed by a β-sandwich structure composed of seven or eight antiparallel β-sheets [224]. The number of small HSPs differs considerably among different species. For instance, a single group is found in yeast, four in *Drosophila* (viz., *HSP22*, *HSP23*, *HSP26* and *HSP27*), ten in humans (*HSPB1* to *HSPB10*), sixteen in *Xenopous laevis*, and more than thirty small HSPs are reported in plants [225,226,227,228,229,230,231]. In *Drosophila*, small HSPs are clustered within 12 kb at locus 67B on chromosome 3L [232,233]. Notably, marine cyanobacteria, such as *Prochlorococus marinus* lack small HSPs in their genomes. Similarly, pathogenic bacteria, such as *Mycoplasma genitalium*, *Haemophilus influenza*, and *Helicobacteria pylori*, also do not possess small HSPs [234,235]. In amphibians, the α-crystalline are not expressed in the lens epithelium of the developing eye [236]. Small HSPs comprise an amino-terminal and a variable C-terminal domain containing 80 to 100 amino acids called the α-crystalline domain [237,238,239]. The α-crystalline domain is abundant in the eye lens protein of vertebrates and the C-terminal domain is involved in the oligomerization complex, which serves as a molecular chaperone [238,240,241]. For example, HSP27 exists as a multimeric complex in the cells and functions similarly to other HSPs in refolding of unfolded proteins (Figure 7). Small HSPs form oligomeric complexes, involving one or more family members, which allow for a large diversity in chaperone specificity [87]. In *Drosophila*, small HSPs such as HSP22, HSP23, HSP26, and HSP27 are present intracellularly and differentially expressed during development. The deletion of the last 42 amino acids in *Drosophila* HSP27 leads to the loss of its high thermotolerance ability [242].

HSP27 is extensively studied in various mammalian tissues, including epithelial cell linings of estrogen-associated targeted sites such as the placenta, connective tissues, and nervous tissues of the female reproductive tract [243]. sHSPs are expressed at basic level, and induced upon high temperature stress or other stressors. HSP27 is also regulated by phosphorylation and dynamic association/dissociation into multimers, comprising dimers or large oligomers. HSP27 has been shown to protect cells from induction of cell death in different ways, including apoptosis, and necrosis upon various physiological stresses [244,245,246]. HSP27 has both pro- and anti-apoptotic functions. It inhibits both intrinsic and extrinsic apoptosis pathways through binding of the unphosphorylated oligomeric form of HSP27 to cytochrome-C and DAXX to the phosphorylated form of HSP27, respectively [229,247]. Besides binding to cytochrome-C, HSP27 also prevents caspase-8-dependent activation of Bid, a pro-apoptotic member of Bcl2 family of proteins. HSP27 interacts with pro-survival Ser/Thr kinases and the microtubule protein actin, and maintaining the integrity of the cytoskeleton, which promotes cell survival [248]. Several human diseases, including cancer, muscle myopathy, cataracts, multiple sclerosis, Alzheimer’s disease, and other neuropathological disorders have been found to be associated with the up- and downregulation of HSP27. HSP27 is indeed implicated in various autoimmune diseases including skin diseases such as pemphigus vulgaris and pemphigus foliaceus, and myasthenia gravis, an autoimmune muscular disorder, and its increased expression is associated with cellular stress and inflammatory processes [249,250]. HSP27 exhibits anti-inflammatory effects, as evidenced by studies showing that reduced expression of HSP27 leads to increased expression of pro-IL-1β and significantly higher release of IL-1β in LPS-treated monocytes [224]. Small HSPs interact with a broad spectrum of cellular substrates and participate in diverse cellular functions and defense mechanisms against various stressors, including high temperature and oxidative stress.

Small HSPs usually form large oligomer complexes that consist of 12–42 subunits [240,251]. These HSPs are the most structurally diverse among the major molecular chaperons, with a monomer size ranging from 12 kDa to 43 kDa. They are characterized by a sequence of about 100 amino acid residues known as the α-crystallin domain [252]. The N-terminal and C-terminal regions of small HSPs are highly variable and are likely responsible for the formation of oligomer assemblies. In humans, the α-crystallin domain is involved in the formation of dimers [253], whereas in *S. cerevisiae*, the HSP26 is localized in cytosol, and forms a dimer when the N-terminal domain is deleted [254]. The quaternary structure of α-crystalline is dynamic, and reflects rapid subunit exchange under normal and stress conditions [255,256]. The subunit exchange reaction is facilitated at high temperatures, which inhibits the aggregation of denatured proteins. In *C. elegans*, the small HSPs, i.e., *HSP12.2*, *HSP12.3*, and *HSP12.6,* exist in both monomer and tetramer forms, which are devoid of chaperone activity. Although oligomerization of small HSPs is important for their distinct function, they may function in monomer form under certain conditions. HSP27 exists in multimeric complex within the cells and participates in chaperonin function to stabilize the denatured and aggregated proteins and refold them to their native form [257]. Phosphorylation of HSP27 results in dissociation of the multimeric complex, which affects its ability to provide stress protection [258,259,260].

Small HSPs are associated with membranes in several mammals. HSPB2, which is expressed in the heart and skeleton muscles, has been shown to associate with the outer membrane of mitochondria [261]. Elevated levels of αβ-crystallin have been found in neurodegenerative diseases such as Alzheimer’s and multiple sclerosis, while a mutation in the αβ-crystallin gene is associated with desmin-related cardiomyopathy and cataractogenesis. A missense mutation (R120G) of αβ-crystallin has been linked to a familial form of desmin-related myopathy (DRM) [262]. Heat shock proteins play an important role in cancer progression, metastasis, and the evasion of apoptosis. HSP90, HSP60, and HSP70 are therapeutically targeted in various cancers. An increased level of HSP27 has also been detected in cancers such as breast cancer, endometrial cancer, and leukemia [55,263,264]. An increased level of HSP27 in breast cancer supports anchorage-independent growth, and increases invasiveness and resistance to chemotherapeutic drugs [265,266,267]. In prostate cancer, the HSP27 level is upregulated after hormonal ablation and is associated with chemotherapy-resistant prostate cancer (Table 4) [268,269]. The cytoprotective function of HSP27 is due to its chaperone functions, direct interference with caspases activation, modulation of oxidative stress, and regulation of cytoskeleton [270,271]. HP27 plays a crucial role in neurodegenerative diseases such as PD. Studies have demonstrated the colocalization of HSP27 with α- synuclein in amyloid fibrils, implicating its involvement in PD pathology. The levels of HSP27 are vital for modulating glycation-associated cellular pathologies in synucleinopathies via its binding and inhibition of amyloid nucleation, fibril binding, and fibril disaggregation [272,273]. As an important regulator of cell survival and its role in different cellular functions in normal and stressful conditions, HSP27 is now considered as an important therapeutic target and biomarker in various human diseases.

### 3.6. Ubiquitin

Ubiquitin are a highly conserved family of 76 amino acid polypeptides and comprise 8 kDa proteins in all eukaryotes. Their expression is induced under stress conditions. Multiple ubiquitin molecules conjugate to form polyubiquitin chains. A chain containing four or more ubiquitin moieties is often necessary for substrate recognition by 26S proteasome complex, facilitating ubiquitin-mediated proteasomal degradation [274,275,276]. The ubiquitin proteasome system (UPS) is responsible for clearance of abnormal denatured protein substrates, as well as facilitating the regulated degradation of short-lived proteins. Ubiquitin molecules play an important role in stress regulation via orchestrating proteasomal disassembly of stress granules. This process leads to the shutdown of cellular pathways and enables the resumption of normal cellular activities, such as nucleocytoplasmic transport and translation, upon recovery of stress [277]. Ubiquitous degradation typically involves three subunits: E1 (ubiquitin-activating enzyme), E2 (ubiquitin-conjugating enzyme), and E3 (ubiquitin ligases) along with deubiquitinase (DUB) enzymes. Ubiquitin residues are essential for the efficient recognition and processing of ubiquitination by 26S proteasome [41,276]. Under certain circumstances, ubiquitination is reversible in signal transduction cascade and protein stabilization processes, facilitated by enzymes known as deubiquitinases (DUBs).

In humans, there are approximately 100 deubiquitinases (DUBs). These can be further divided into seven sub-families: ubiquitin C-terminal hydrolase (UCHs), ubiquitin specific proteases (USPs), ovarian tumor proteases (OTUs), Josephins JAB1/MINDY/ZUFSP, and six (except JAMNs) belongs to the Cys proteases, while the JAMN family is composed of zinc-dependent metalloproteases (JAMMs, also known as MPN^+^) [278]. DUB activity is highly specific, characterized by multiple level regulations that distinguish it among many ubiquitin-like molecules, isopeptides, and linear peptides, and between different types of ubiquitin linkage and chain structures. An USP29 binds and cleaves the polyubiquitin chain from p53 upon oxidative stress, thus coordinating the molecular and cellular stress response from oxidative stress [279]. Further, it has also been shown that inhibition of USP14 exaggerates degradation of several proteasome substrates [280,281]. The unfolded and damaged proteins are recognized by the ubiquitin–proteasome pathway and maintain protein quality control (PQC). These undesired proteins are targeted by chaperone-bound ubiquitin E3 enzymes, such as STIP1 homology and the u-Box-containing protein1 (CHIP), BAG co-chaperone 1 (BAG1), and scythe [282]. Polyubiquitin conjugation has largely been attributed to increased protein quality control (PQC) activity in response to stress-induced protein damage and translational arrest [283]. In recent years, studies have demonstrated the pivotal role of USPs in regulating ER stress in neuronal cells. In Huntington’s disease, USP14 regulate ER stress-mediated cell death through IRE1, so USP14 may be a potential target for future treatment of HD and other cluster diseases [284]. USP14 is also reported in regulating metabolic disease such as obesity and type 2 diabetes (T2DM). Glucose homeostasis is impaired in obesity, results in induced ER stress, and increases USP14 transcription level [285]. Knockdown USP14 in obese mice showed reduced glucose, and ER stress suggested USP14 has potential targets for metabolic disease therapeutics. In neurodegeneration, protein aggregates and unfolded protein-induced ER stress have significant role in the pathogenesis of neurodegenerative diseases, such as Alzheimer’s disease, amyotrophic lateral sclerosis (ALS), Parkinson’s disease (PD), and hypermetabolic diseases. In Parkinson’s disease, parkin, a ubiquitin E3 ligase, is a crucial target involved in various cellular process associated with the diseases [286]. Parkin targets for ubiquitination include parkin-interacting substrate (PARIS), which accumulates in the brains of patients with autosomal recessive juvenile PD [287]. A mutation in parkin leads to the accumulation of PARIS, subsequently inhibiting the transcription of peroxisome proliferator-activated receptor gamma coactivator 1-alpha (PGC-1α) and its downstream targets. This affects the degradation of damaged mitochondria and biogenesis of mitochondria. Mutations in parkin are linked to autosomal recessive juvenile parkinsonism (AR-JP) [288]. It has been reported that accumulation of misfolded α-synuclein (α-syn) leads to the UPS dysfunction in dopaminergic neurons in vivo, particularly in early stages of PD [138,289]. In this scenario, HSPs play a role in clearing aggregates by activating both autophagy and proteasomal degradation pathways [290,291]. In cancer, the protein kinase RNA-like ER kinase (PERK) is associated with both the ubiquitin-proteasome system (UPS) and endoplasmic reticulum (ER) stress. PERK levels are significantly elevated in various cancers, including kidney renal papillary cell carcinoma, lower-grade glioma of the brain, invasive breast carcinoma, thyroid carcinoma, and neck squamous cell carcinoma. PERK-mediated upregulation of vascular endothelial growth factor (VEGF), fibroblast growth factor-2 (FGF2), and interleukin-6 (IL-6), coupled with the downregulation of anti-angiogenic cytokines, markedly promotes tumor growth [292] (Table 4). Therefore, UPS regulates essential proteins involved in various cellular processes in response to stress, including Wnt/β-catenin, HIF-α, and p53, thereby influencing tumorigenesis [282]. Thus, targeting UPS and proteasomal pathways could be a promising approach to understanding the underlying molecular mechanism of neurodegenerative diseases, cancer, and age-related problems. To develop an effective treatment using UPS and degradative pathways would aid in improving the quality of life for millions of people worldwide.

**Table 4:** HSPs and their potential role in in various human diseases.

Diseases	Stress Proteins	Role of Stress Proteins	References
Cancers	HSP90	Affecting client protein interaction	[107,108,127,128]
	HSP70	Promoting cell survival	[150,155,156,171,255]
	HSP60,	Interact with cytochrome-C and DAXX pro- and anti-apoptotic role	[220]
	sHSPs	Anchorage-independent growth, increase invasiveness	[256,257,258]
	Ubiquitin	Regulating ER stress, PERK-mediated UPS	[273,283]
Neurodegeneration, dementia, Alzheimer’s disease, Parkinson’s disease	HSP100	Binding with HSP70 and HSP40 and preventing aggregates	[105,107,108]
	HSP90	Cdc37 complex disruption,	[135,136]
	HSP70	Mitochondrial integrity, oxidative stress	[170]
	HSP60	Pro-inflammatory cytokines IL-1β, IL-6, and TNF-α, binds to Aβ oligomers	[205,213]
	HSP27	αβ-crystallin, α- synuclein	[263,264]
	Ubiquitin	Parkin, a ubiquitin E3 ligase, misfolded α-synuclein	[138,287]
Auto-immune disease	HSP60	HSP60 peptides, elicit cytotoxic T cell responses	[195,196,197,198]
	HSP27	Cellular stress, 1L-1β in LPS-treated monocytes	[216,240,241]
Infectious diseases	HSP70	Viral replication	[158,159,160,161]
	HSP60	Cell surface expression IL8	[202]
Inflammation Rheumatoid arthritis	HSP60	Cytokine signaling processes and release	[206,207,208]
Cardiovascular disease	HSP70	Insulin resistance and anti-inflammatory effect	[170]
	HSP60	TLR2 and TLR4 functions	[193]
	HSP27	Desmin-linked	[253]
Metabolic diseases Diabetes	HSP70	Increases sensitivity to insulin	[165,166,167,171]
	HSP60	Modulates the TLRs and IGF-I receptor level, PI3-K/Akt activation	[196,197,198,199,200]
	Ubiquitin	IRE1, USP14-mediated regulation	[274,275]

## 4. Conclusions and Future Directions

This review explores the expression pattern of HSP-related genes/proteins and their role in protection of cells against various insults such as high temperature, oxidative stress, protein aggregation, and degeneration. During stress conditions, stress response factors are upregulated, leading to the oligomerization of HSFs, their translocation to nucleus, binding to the heat shock elements (HSEs) in the promoter, and induction of transcription of heat shock genes. While these cellular changes are adaptive in the short term, they require coordinated reversal after stress is removed to resume cellular activities and reestablish homeostasis. In response to stress-induced protein damage and translational arrest, ribosome-free mRNA is marked by polyubiquitin conjugates to ubiquitination to resume normal cellular activity and maintain protein quality control [277,283]. Stress proteins are highly induced by pesticides, and environmental toxicants [293]. HSPs play a critical role in maintaining protein homeostasis, which is essential for cell integrity, survival, and metabolism. Impairment of chaperone-aided protein quality control can lead to the onset and progression of various diseases.

HSPs also play a dual role in regulating cell apoptosis and cell death. HSP70 inhibits cytochrome c release by forming the Bax-HSP70-HSp40 complex, attenuating BAX mitochondria translocation. HSP90 and HSP70 can attenuate apoptosis by stabilizing and activating AKT signaling pathways, which facilitate cancer cell survival [294]. The expression of HSP60 remains unaltered even after treatment of transcriptional and translational inhibitors, like actinomycin–D and cycloheximide, suggesting that HSP60 expression is regulated differently [179]. The thermal sensitivity of cells is affected by different environmental factors, such as pH [295], elevated temperature [296], cold shock [297], heavy metals [92,298,299,300], etc., which require induced transcription of HSP as a protective mechanism to maintain the cellular homeostasis. Alternatively, the thermal sensitivity or the stress response of different cell types may vary with physiological state or the inducer, which ultimately influences the expression pattern of responsive gene/s. Heat shock-induced ubiquitination primes the cell for recovery from stress by targeting specific proteins involved in several pathways downregulated during stress. Further investigation is warranted to delve into the role of HSPs in the development of potential therapeutics for a range of human diseases characterized by severe stress conditions and disruption of protein homeostasis.

## Figures and Tables

**Table 2:** Classification of heat shock proteins based on their molecular weight.

Name	Size (kDa)	Bacterial Homolog	Location	Functions
HSP 100	104/110	Clp	Cytosol, nucleus	Mitigate severe stress
HSP 90	90	HtpG	Cytosol, nucleus	Part of the steroid hormone receptor complex; stabilize substrate proteins; and inhibit protein aggregation
HSP 70	72	Dna K	Cytosol, nucleus	Highly stress inducible
HSC 70	73	Dna K	cytosol	Constitutively expressed
HSP 60	60	GroEL	Mitochondria, chloroplast, and nucleus	Assists protein folding
HSP 40	47	Dna J	Endoplasmic reticulum	Co-chaperone of Dna K; protein folding and refolding
Small HSPs	20–34	IbpA/B	Cytosol, nucleus	Prevent aggregation of proteins
HSP10	10	GroES	mitochondria, chloroplast	Assist as a co-chaperone
Ubiquitin	8		Cytosol, nucleus	Involved in non-lysosomal protein degradation

**Table 3:** List of molecular chaperones, co-chaperones, their localization and functions.

Chaperonin	Organism	Chaperone	Co-Chaperone	Localization	Functions
HSP60/ HSPD1	Bacterial	GroEL	GroES	Cytosol	Assist folding and refolding of denatured proteins
	Mammalian	mHSP60 (HSPD)/ TriC/CCT	HSP10 (HSPE)	Mitochondria, cytosol	Folding of nascent proteins and mitochondria proteostasis
HSP40/HSPF	Bacterial	DnaJ	DnaK/GrpE	Cytosol	Modulating activity of DnaK, associated with nascent polypeptides, binds to unfolded proteins
	Mammalian	Hdj1/2, HSP40, auxilin	HSP70/HIP	Cytosol	Modulating ATPase activity of DnaK, auxilin recruits HSP70 partner HSC70 to uncoat clathrin-coated vesicles
HSP70/HSPA	Bacterial	DnaK	DnaJ/GrpE/ClpB	Cytosol	Folding and export of nascent peptides, disaggregation and degradation of stress-induced folding and translocation
	Mammalian	Bip/Grp78	DnaJ-like ER proteins (Grp70, Sil1/sls1)	Endoplasmic reticulum	Involved in calcium homeostasis, translocation, folding, transport and re-translocation of polypeptides, regulation of unfolded protein response
		HSC70 (HSP73), HSP70 (HSP72)	HSP40, Hop, Bag1-5, HIP, HSPBP1, CHIP, SGT, HSP110, Homologs to Tom 70, TPR1	Cytosol	Folding and transportation of nascent polypeptide, inhibits mis-folding and aggregations
		mHSP70/Grp75/mortalin	-	Cytosol	Protein folding and translocation into mitochondria
HSP90/HSPC	Bacterial	HtpG	-	Cytosol	Stress-responsive protein folding
	Mammalian	HSP90/83/89, TRAP1/2	HOP/HIP, HSP70, p50, p23, CHIP, Sgt1/TPR2, Immunophilins	Cytosol, mitochondrial	Folding and conformational regulation of signaling protein, regulation of steroid hormone receptor and kinases
		Grp94	Grp78	ER	Folding and assembly of secretary proteins
HSP100/HSPH	Bacterial	ClpA	ClpP, SspB	Cytosol	ATP dependent protein unfolding and proteolysis
		ClpB	Dnak, DnaJ, GrpE	Cytosol	ATP dependent processing of aggregated proteins.
Small HSPs (HSPB)	Bacterial	IbpA/IbpB	-	Cytosol	Associated with inclusion bodies, prevent heat denatured protein aggregation
	Mammalian	α-crystallin, HSP27	-	Cytosol	Prevent heat denatured protein aggregation, regulate microfilament polymerization
Chaperones	Bacterial	HSP33, SecB	SecA	Cytosol	Prevent aggregation of oxidative damage proteins. Shuttling of secretory proteins SecA/B, maintenance of periplasm proteins, Pili assembly
		SKP/PapD/FimC	PapC, FimD	Periplasm	
	Mammalian	Calnexin, calreticulin, PDI, HSP47 (collagen)	ERp57, Cnx/Crt	Endoplasmic reticulum (ER)	Folding of ER glycosylated proteins (Cnx/Crt); collagen biosynthesis (HSP47), assist di-sulfide bond formation.

## Data Availability

Not applicable.
